# Reference ranges and dimensional structure of upper body posture parameters in adults: a cross-sectional study

**DOI:** 10.3389/fresc.2026.1930857

**Published:** 2026-09-30

**Authors:** Lianhua Yin, Chendong Ruan, Xiaojin Zhuang, Lijuan Wu, Xuling Ni, Jing Tao, Lidian Chen

**Affiliations:** 1 Health Management Center, Second Affiliated Hospital of Fujian University of Traditional Chinese Medicine, Fuzhou, Fujian, China; 2 College of Rehabilitation Medicine, Fujian University of Traditional Chinese Medicine, Fuzhou, Fujian, China; 3 National-Local Joint Engineering Research Center of Rehabilitation Medicine Technology, Fujian University of Traditional Chinese Medicine, Fuzhou, Fujian, China; 4 Fujian Key Laboratory of Rehabilitation Technology, Fujian University of Traditional Chinese Medicine, Fuzhou, Fujian, China

**Keywords:** exploratory graph analysis, reference values, scoliosis screening, surface topography, upper body posture

## Abstract

**Background:**

Upper body posture assessment using surface topography can support population-level screening, but adult reference data for these parameters remain limited, restricting clinical interpretability. This study aimed to establish reference ranges for upper body posture parameters in adults and characterize their underlying dimensional structure.

**Methods:**

This cross-sectional study analyzed DIERS three-dimensional surface topography data from 1,859 adults (mean age 46.9 ± 11.9 years; 52.7% male). Descriptive statistics were generated for 45 DIERS-derived parameters spanning six measurement domains. Participants were classified into three screening categories of scoliotic posture, scoliosis risk, and unclassified, based on pelvic obliquity, vertebral rotation, and apical vertebral deviation. Group differences by age, sex, and screening category were examined using ANOVA and *t*-tests with false discovery rate correction; effect sizes were quantified with *η*^2^ and Cohen's *d*. Exploratory graph analysis (EGA) with 1,000-iteration bootstrap validation identified the dimensional structure of the retained parameters.

**Results:**

The screening category distribution was scoliotic posture 35.9%, scoliosis risk 5.8%, and unclassified 58.4%, with no significant variation by age or sex. Sex-related differences were pronounced: 13 of 31 significantly different parameters showed large effects (Cohen's *d* ≥ 0.80), concentrated in distance measurements, postural reference points, spinal curve angles, and shoulder parameters. Age-related between-group differences were observed across multiple parameters, but effect sizes were predominantly trivial to small (*η*^2^ < 0.06). EGA identified a stable five-dimension structure (bootstrap support: 98.5%): trunk size and segmental length, coronal deviation and axial rotation, sagittal spinal structure and postural balance, upper-body angular asymmetry, and pelvic asymmetry.

**Conclusions:**

This study established adult population reference ranges for upper body posture parameters derived from surface topography. The analysis revealed pronounced sex-related differences, suggesting that sex-stratified reference values may be more appropriate for clinical interpretation. Five distinct dimensions of upper body posture parameters were identified, indicating that future research should consider coordinated changes across multiple anatomical structures. Together, these findings provide a quantitative basis for interpreting surface topography results in health management and scoliosis screening.

## 1 Introduction

The spine is essential for load bearing and postural control, and its morphology and alignment are closely related to spinal health across the life course. Scoliosis represents an important form of spinal morphological alteration (1). Adolescent idiopathic scoliosis affects up to 3% of the population (2, 3), whereas adult degenerative scoliosis is common in older adults and becomes more prevalent with increasing age (4, 5). Scoliosis may be associated with pain and reduced health-related quality of life, particularly as deformity severity increases (6). Early identification is therefore important for supporting preventive and rehabilitative care (7–9).

Radiographic examination is the gold standard for diagnosing scoliosis and quantifying curve magnitude (10). However, repeated radiographic assessment is difficult for individuals who require ongoing evaluation because of cost and cumulative radiation exposure (11, 12). Public health and health management settings therefore need a radiation-free, easily deployable assessment method that can provide preliminary information on spinal morphology before diagnostic imaging is considered (13, 14).

Three-dimensional surface topography systems, such as DIERS (15, 16), provide a feasible approach to this need. Based on video raster stereography, these systems reconstruct a three-dimensional model of the back surface from the curvature of projected raster lines. They generate quantitative parameters of upper body posture and spinal morphology, such as trunk length and indicators of spinal alignment. Previous studies have provided evidence for the reliability and validity of this approach, Degenhardt et al. reported good test-retest reliability for multiple DIERS Formetric surface topography parameters, with intraclass correlation coefficients of 0.75 or higher in 26 of 40 parameter groups (17). Meta-analytic evidence has also shown strong correlations between surface topography parameters and radiographic Cobb angle (*r* > 0.8) (18, 19). Taken together, these findings support surface topography as a complementary tool for scoliosis screening and postural assessment (20).

Despite these advances, important interpretive gaps remain. Previous studies have reported reference values for upper body posture parameters in adults using different surface topography systems (21, 22), and DIERS based criteria have also been proposed to identify scoliosis related postural patterns (23, 24). Together, these studies provide important evidence for interpreting surface topography measurements and their clinical relevance. However, such evidence remains limited in East Asian adult populations, restricting the interpretation and application of surface topography assessment in health management and scoliosis screening (20, 25).

Surface topography generates multiple posture parameters that may contain shared or overlapping information. Ohlendorf et al. used factor analysis to identify common factors underlying upper body posture parameters in healthy adults (22), while Manca et al. used correlation and principal component analysis to identify representative patterns among interrelated raster-stereographic parameters in individuals with scoliosis (26). These findings suggest that upper body posture parameters may exhibit an underlying structure.

Therefore, the primary aim of this study was to establish reference values for upper body posture parameters in East Asian adults. The secondary aim was to characterize the underlying structure of these parameters.

## 2 Materials and methods

### 2.1 Data source

This study used data from a database of three-dimensional spinal posture assessments conducted at the Second Affiliated Hospital of Fujian University of Traditional Chinese Medicine between 2014 and 2021. Individuals who provided informed consent and were able to complete the DIERS assessment were included in the database. The technical exclusion criteria were: 1) BMI >37 kg/m^2^; 2) very dark skin pigmentation; 3) extensive scars or tattoos on the back; 4) adhesive dressings, medicated patches, or bandages on the back; and 5) excessive back hair. No additional clinical eligibility screening was applied, and conditions such as known spinal pathology, prior spinal surgery, pregnancy, or acute back pain were not used as exclusion criteria.

### 2.2 Ethical considerations

This study was approved by the Medical Research Ethics Committee of the Second Affiliated Hospital of Fujian University of Traditional Chinese Medicine (Approval No. SPHFJP-K2019059-02). All data used for analysis were de-identified before access by the research team, and no personally identifiable information was included in the analytic dataset or reported results.

### 2.3 DIERS surface topography assessment

All participants underwent spinal morphology assessment using the DIERS system (RS Scan, Beijing, China) (15). Assessments were performed by examiners who had received training in the DIERS assessment procedures. Participants removed all clothing except their underwear and stood on the testing platform. The examiner placed markers on the bilateral acromion points. Participants were instructed to lower the back waistband of their underwear to expose part of the intergluteal cleft, maintain their usual natural standing posture, keep both arms relaxed and hanging naturally, lower the head slightly by approximately 15°, place both feet apart with the heels aligned, and remain as still as possible. The examiner adjusted the grating height to ensure that the light projection area covered the region from above the neck to below the buttocks. Each acquisition lasted 6 s. All frames were reviewed after acquisition, and frames with inadequate quality were excluded. The assessment was repeated when excessive body movement occurred. The automatically identified anatomical landmarks were checked for consistency with clinical standards. If automatic recognition failed, the landmarks were manually adjusted. The final parameter values were calculated by averaging the retained valid frames.

### 2.4 Upper body posture parameters and screening categories

Previous studies reported 40 DIERS-generated parameters (17), whereas our study included 45 parameters generated by the DIERS system, with five additional shoulder-related parameters. These parameters covered six domains: Length Measurements, Trunk and Pelvis Imbalances, Location of Postural Reference Points, Spinal Curve Angles, Spinal Deviation, and Shoulder-related Parameters. The definitions and units of these parameters are provided in Table 1. Although some upper body posture constructs were represented by multiple related parameters, all parameters were retained and reported separately to facilitate reproducibility. To facilitate clinical interpretation of the population reference values, participants were further classified according to previously reported DIERS criteria for scoliosis risk (23, 24). These categories were based on surface topography parameters and were not considered equivalent to a radiographic diagnosis of scoliosis. Participants were categorized as scoliotic posture when absolute pelvic obliquity DL DR was ≤5 mm, vertebral rotation RMS was ≤5°, and apical vertebral deviation VP-DM RMS was ≤5 mm. Participants were categorized as scoliosis risk when pelvic obliquity DL-DR was >5 mm, vertebral rotation RMS was >5°, and apical vertebral deviation VP-DM RMS was >5 mm. All other combinations were categorized as unclassified.

**Table 1:** Definitions of DIERS-derived parameters.

Parameter	Unit	Definition
Length measurements		
Trunk length VP–DM	mm	Length from the vertebra prominens (VP) to the dimple midpoint (DM), defined as the midpoint between the left and right lumbar dimples.
Trunk length VP–SP	mm	Length from VP to the sacral point (SP).
VP–SP/VP–DM	%	Ratio of the VP–SP length to the VP–DM length.
Dimple distance DL–DR	mm	Distance between the left lumbar dimple (DL) and the right lumbar dimple (DR).
DL–DR/VP–DM	%	Ratio of the DL–DR distance to the VP–DM length.
Trunk length T4–DM	mm	Length from the fourth thoracic vertebra (T4) to DM.
Trunk and pelvis imbalances		
Sagittal imbalance VP–DM^a^	°	Angle between the VP–DM line and the external vertical reference line in the sagittal plane.
Sagittal imbalance VP–DM^a^	mm	Horizontal offset between VP and DM in the sagittal plane.
Coronal imbalance VP–DM^b^	°	Angle between the VP–DM line and the vertical reference line in the frontal plane.
Coronal imbalance VP–DM^b^	mm	Lateral offset between VP and DM in the frontal plane.
Pelvic obliquity DL–DR^c^	°	Angle between the DL–DR line and the horizontal reference line in the frontal plane.
Pelvic obliquity DL–DR^c^	mm	Vertical offset between DL and DR in the sagittal plane.
Pelvic torsion DL–DR^d^	°	Relative torsion angle between the DL and DR surface normal when the left and right sides of the pelvis rotate in opposite directions around a horizontal axis passing through the pubic symphysis.
Pelvic inclination (dimples)^e^	°	Mean sagittal-plane angle between the surface normal vectors at DL and DR and the horizontal reference line.
Pelvic inclination (symmetry line)^e^	°	Sagittal-plane angle between the surface normal vector at DM on the spinal symmetry line and the horizontal reference line.
Pelvis rotation^f^	°	Rotation angle of the pelvis around the vertical axis, calculated from the difference in the anterior-posterior positions of DL and DR.
Location of postural reference points		
Cervicothoracic inflection point (ICT)^g^	mm	Vertical distance from ICT to the projected VP point on the vertical reference line passing through KA.
Kyphotic apex (KA)^g^	mm	Vertical distance from KA to the projected VP point on the vertical reference line passing through KA.
Thoracolumbar inflection point (ITL)^g^	mm	Vertical distance from ITL to the projected VP point on the vertical reference line passing through KA.
Lordotic apex (LA)^g^	mm	Vertical distance from LA to the projected VP point on the vertical reference line passing through KA.
Lumbosacral inflection point (ILS)^g^	mm	Vertical distance from ILS to the projected VP point on the vertical reference line passing through KA.
Flèche cervicale	mm	Horizontal distance from the cervical flèche point to the vertical reference line passing through KA.
Flèche lombaire	mm	Horizontal distance from the lumbar flèche point to the vertical reference line passing through KA.
Spinal curve angles		
Kyphotic angle ICT–ITL	°	Angle formed by the surface tangent lines at ICT and ITL in the sagittal back profile.
Kyphotic angle VP–ITL	°	Angle formed by the surface tangent lines at VP and ITL in the sagittal back profile.
Kyphotic angle VP–T12	°	Angle formed by the surface tangent lines at VP and T12 in the sagittal back profile.
Lordotic angle ITL–ILS	°	Angle formed by the surface tangent lines at ITL and ILS in the sagittal back profile.
Lordotic angle ITL–DM	°	Angle formed by the surface tangent lines at ITL and DM in the sagittal back profile.
Lordotic angle T12–DM	°	Angle formed by the surface tangent lines at T12 and DM in the sagittal back profile.
Spinal deviation		
Vertebral rotation (RMS)	°	Root mean square (RMS) of vertebral rotation values derived from the horizontal components of the surface normal vectors along the reconstructed spinal symmetry line.
Vertebral rotation (max)	°	Maximum vertebral rotation value along the reconstructed spinal symmetry line, with the sign indicating direction.
Vertebral rotation (+max)^h^	°	Largest positive vertebral rotation value along the reconstructed spinal symmetry line, representing maximum rightward rotation.
Vertebral rotation (−max)^h^	°	Largest negative vertebral rotation value along the reconstructed spinal symmetry line, representing maximum leftward rotation.
Vertebral rotation (amplitude)	°	Difference between the maximum positive and maximum negative vertebral rotation values along the reconstructed spinal symmetry line.
Trunk torsion^i^	°	Angle between the surface normal vectors at VP and DM in the horizontal plane.
Apical deviation VP–DM (RMS)	mm	RMS deviation of the reconstructed spinal midline from the VP–DM reference line in the frontal plane.
Apical deviation VP–DM (max)	mm	Maximum deviation of the reconstructed spinal midline from the VP–DM reference line in the frontal plane, with the sign indicating direction.
Apical deviation VP–DM (+max)^j^	mm	Largest positive deviation of the reconstructed spinal midline from the VP–DM reference line, representing maximum rightward deviation.
Apical deviation VP–DM (−max)^j^	mm	Largest negative deviation of the reconstructed spinal midline from the VP–DM reference line, representing maximum leftward deviation.
Apical deviation VP–DM (amplitude)	mm	Difference between the maximum positive and maximum negative deviations of the reconstructed spinal midline from the VP–DM reference line.
Shoulder-related parameters		
Shoulder distance SL–SR	mm	Distance between the left shoulder point (SL) and the right shoulder point (SR).
Shoulder tilt SL–SR^k^	°	Angle between the SL–SR line and the horizontal reference line in the frontal plane.
Shoulder tilt SL–SR^k^	mm	Vertical height difference between SL and SR.
Shoulder rotation SL–SR (pelvis) ^l^	°	Difference between the transverse-plane rotation of the shoulder line and the transverse-plane rotation of the pelvis.
Shoulder rotation SL–SR (baseline)^l^	°	Angle of the shoulder line relative to the system reference coronal plane in the transverse plane.

Directional signs follow the DIERS output convention: ^a^positive values indicate posterior inclination and negative values indicate anterior inclination; ^b^positive values indicate VP right of DM and negative values indicate VP left of DM; ^c^positive values indicate DR higher than DL and negative values indicate DR lower than DL; ^d^positive values indicate DR surface normal more upward than DL surface normal and negative values indicate the opposite direction; ^e^positive values indicate posterior pelvic inclination and negative values indicate anterior pelvic inclination; ^f^positive values indicate pelvic rotation toward the participant's right side and negative values indicate rotation toward the participant's left side; ^g^positive values indicate positions above VP and negative values indicate positions below VP; ^h^positive values indicate rightward rotation and negative values indicate leftward rotation; ^i^positive values indicate rightward orientation of the VP surface normal and negative values indicate leftward orientation; ^j^positive values indicate rightward spinal deviation and negative values indicate leftward spinal deviation; ^k^positive values indicate SR higher than SL and negative values indicate SR lower than SL; ^l^positive values indicate SR anterior to SL and negative values indicate SR posterior to SL.

### 2.5 Statistical analysis

Statistical analyses were performed using SPSS software (version 28.0; IBM Corp., Armonk, NY, USA). Continuous variables were described as mean ± standard deviation and median (interquartile range), and categorical variables were described as frequencies and percentages. The distributions of sex and age group across the screening categories were compared using the chi square test, and age trends in screening category proportions were further assessed using Cochran Armitage trend tests.

The 45 DIERS-derived parameters were summarized in the total sample and compared across age groups, sex, and DIERS based screening categories. Homogeneity of variance was assessed using Levene's test (27). Differences across age groups and screening categories were examined using Welch's one-way ANOVA (28), whereas sex-related differences were examined using Welch's independent-samples *t*-tests. Effect sizes for age- and screening-category comparisons were quantified using eta squared (*η*^2^), and sex-related differences were quantified using Cohen's *d*. *η*^2^ was interpreted as trivial (<0.01), small (0.01–0.06), medium (0.06–0.14), or large (≥0.14). Cohen's d was interpreted as negligible (<0.20), small (0.20–0.50), medium (0.50–0.80), or large (≥0.80) (29, 30). *P* values were adjusted using the Benjamini Hochberg false discovery rate procedure separately within the 45 comparisons for age, sex, and screening category (31). Age was additionally examined as a continuous variable with adjustment for sex in a sensitivity analysis. EGA was performed in R (version 4.6.0) using the EGAnet package (version 2.4.1) to explore the clustering structure of the DIERS-derived parameters (32). The stability of the EGA solution was evaluated using bootstrap EGA with 1,000 iterations (33).

## 3 Results

### 3.1 Participant characteristics and screening category distribution

A total of 1,859 adult participants were included, with a mean age of 46.9 ± 11.9 years. The sample included 980 men (52.7%) and 879 women (47.3%). According to the DIERS-derived screening classification, 667 participants (35.9%) were classified as scoliotic posture, 107 (5.8%) as scoliosis risk, and 1,085 (58.4%) as unclassified (Table 2). The overall distribution of screening categories did not differ significantly by sex or age group (*P* = 0.271 and *P* = 0.110, respectively; Table 3). Further trend analysis showed that the proportion classified as scoliosis risk increased across age groups, from 4.3% in participants aged 18–39 years to 5.9% in those aged 40–59 years and 8.1% in those aged ≥60 years (adjusted *P* = 0.049), whereas no significant age trends were observed for scoliotic posture or unclassified categories (adjusted *P* = 0.132 and 0.599, respectively; Supplementary Table S1).

**Table 2:** Characteristics of study participants.

Characteristic	Total *N* = 1,859
Age, years, mean ± SD	46.9 ± 11.9
Sex, *n* (%)	
Male	980 (52.7)
Female	879 (47.3)
Age group, *n* (%)	
18–39 years	628 (33.8)
40–59 years	911 (49.0)
≥60 years	320 (17.2)
Screening classification, *n* (%)	
Scoliotic posture	667 (35.9)
Scoliosis risk	107 (5.8)
Unclassified	1,085 (58.4)

**Table 3:** Distribution of screening categories by sex and age group.

Characteristic	Scoliotic posture, *n* (%)	Scoliosis risk, *n* (%)	Unclassified, *n* (%)	*χ* ^2^	*P*
Sex					
Male (*n* = 980)	336 (34.3)	55 (5.6)	589 (60.1)	2.61	0.271
Female (*n* = 879)	331 (37.7)	52 (5.9)	496 (56.4)		
Age group					
18–39 years (*n* = 628)	238 (37.9)	27 (4.3)	363 (57.8)	7.54	0.110
40–59 years (*n* = 911)	326 (35.8)	54 (5.9)	531 (58.3)		
≥60 years (*n* = 320)	103 (32.2)	26 (8.1)	191 (59.7)		

Percentages are calculated within each sex or age group. *χ*^2^, chi-square test statistic; *P* < 0.05 was considered statistically significant.

### 3.2 Reference values for DIERS-derived parameters

Descriptive statistics for the 45 DIERS-derived parameters are shown in Table 4. Comparisons with directly comparable parameters from previously published adult reference datasets are provided for the overall sample and by sex (Supplementary Tables S2, S3, respectively). Among the three parameters used for screening classification, the mean vertebral rotation RMS was 3.69 ± 1.70°, the mean apical deviation VP-DM RMS was 4.90 ± 2.85 mm, and the mean pelvic obliquity DL-DR was 0.42 ± 9.38 mm. The corresponding medians were 3.43° (IQR, 2.56–4.48), 4.25 mm (IQR, 2.95–6.09), and 0.00 mm (IQR, −3.00 –3.00), respectively.

**Table 4:** Descriptive statistics and reference limits for DIERS-derived parameters.

Parameter	Unit	Mean ± SD	Median (IQR)	2.5th percentile	97.5th percentile
Distance measurements					
Trunk length VP–DM	mm	465.38 ± 33.52	465.42 (442.54–487.96)	402.36	530.17
Trunk length VP–SP	mm	519.78 ± 32.98	519.61 (496.77–541.50)	459.54	584.18
VP–SP/VP–DM	%	111.79 ± 2.76	111.26 (110.34–112.59)	107.48	118.64
Dimple distance DL–DR	mm	92.94 ± 13.41	93.19 (87.00–102.01)	60.76	114.18
DL–DR/ VP–DM	%	20.06 ± 3.35	20.01 (18.18–21.99)	13.7	25.84
Trunk length T4–DM	mm	386.37 ± 27.68	386.72 (367.17–405.03)	334.39	439.5
Trunk and pelvis imbalances					
Sagittal imbalance VP–DM	°	0.88 ± 2.43	0.88 (−0.64–2.37)	−4.06	5.78
Sagittal imbalance VP–DM	mm	7.54 ± 19.82	7.23 (−5.22–19.66)	−30.92	48.25
Coronal imbalance VP–DM	°	−0.55 ± 1.43	−0.55 (−1.39–0.32)	−3.4	2.46
Coronal imbalance VP–DM	mm	−4.48 ± 11.57	−4.50 (−11.38–2.69)	−27.48	19.5
Pelvic obliquity DL–DR	°	0.23 ± 5.02	0.00 (−1.91–2.05)	−8.13	8.37
Pelvic obliquity DL–DR^‡^	mm	0.42 ± 9.38	0.00 (−3.00–3.00)	−12	12
Pelvic torsion DL–DR	°	0.55 ± 3.24	0.89 (−1.30–2.31)	−5.1	5.59
Pelvic inclination (dimples)	°	16.09 ± 6.35	16.70 (12.02–20.55)	2.69	26.52
Pelvic inclination (symmetry line)	°	16.32 ± 7.96	16.39 (10.97–21.73)	1.26	31.78
Pelvis rotation	°	−0.67 ± 3.38	−0.30 (−2.78–1.28)	−7.27	6.05
Location of postural reference points					
ICT	mm	6.25 ± 9.77	7.64 (1.51–13.10)	−18.9	19.58
KA	mm	−165.48 ± 43.48	−169.88 (−195.08–-140.56)	−241.92	−61.77
ITL	mm	−312.22 ± 40.80	−308.72 (−340.53–-281.62)	−393.44	−243.37
LA	mm	−396.27 ± 41.69	−395.02 (−422.46–-365.93)	−482.76	−323.98
ILS	mm	−471.09 ± 39.21	−472.72 (−499.67–−442.96)	−541.17	−398.12
Flèche cervicale	mm	60.97 ± 19.61	59.56 (47.53–73.50)	27.02	101.74
Flèche lombaire	mm	40.83 ± 12.46	40.41 (31.75–48.90)	18.5	67.29
Spinal curve angles					
Kyphotic angle ICT–ITL	°	50.17 ± 8.89	49.85 (44.46–55.58)	32.8	69.12
Kyphotic angle VP–ITL	°	46.36 ± 8.68	46.01 (40.62–51.85)	29.92	63.83
Kyphotic angle VP–T12	°	42.58 ± 7.81	42.61 (37.63–47.62)	26.87	58.18
Lordotic angle ITL–ILS	°	36.49 ± 8.75	36.37 (30.41–42.12)	20.24	54.82
Lordotic angle ITL–DM	°	32.37 ± 8.73	32.19 (26.41–38.10)	15.96	50.48
Lordotic angle T12–DM	°	28.58 ± 9.09	28.62 (22.55–34.27)	12.09	46.67
Spinal Deviation					
Vertebral rotation (RMS)^‡^	°	3.69 ± 1.70	3.43 (2.56–4.48)	1.37	7.61
Vertebral rotation (max)	°	0.46 ± 8.00	−2.45 (−6.46–7.61)	−11.23	13.67
Vertebral rotation (+max)	°	4.91 ± 4.12	4.21 (1.33–7.89)	−0.52	13.67
Vertebral rotation (−max)	°	−4.88 ± 3.20	−4.55 (−6.83–−2.52)	−11.25	0.08
Vertebral rotation (amplitude)	°	9.79 ± 4.12	9.22 (6.73–12.10)	3.72	19.92
Trunk torsion	°	1.15 ± 5.45	1.14 (−1.99–4.01)	−9.09	12.63
Apical deviation VP–DM (RMS)^‡^	mm	4.90 ± 2.85	4.25 (2.95–6.09)	1.51	11.72
Apical deviation VP–DM (max)	mm	3.82 ± 9.31	6.20 (−5.00–9.81)	−12.94	19.1
Apical deviation VP–DM (+max)	mm	7.22 ± 5.32	6.44 (3.55–9.88)	0	19.1
Apical deviation VP–DM (−max)	mm	−4.05 ± 3.83	−3.21 (−5.83–−1.15)	−12.98	0
Apical deviation VP–DM (amplitude)	mm	11.27 ± 5.12	10.34 (7.86–13.78)	4.43	22.83
Shoulder-related parameters					
Shoulder distance SL–SR	mm	307.66 ± 23.36	307.60 (291.61–322.83)	264.1	355.65
Shoulder tilt SL–SR	°	−0.17 ± 1.45	−0.16 (−1.01–0.61)	−3.09	2.9
Shoulder tilt SL–SR	mm	−0.95 ± 7.81	−0.90 (−5.49–3.26)	−16.64	15.08
Shoulder rotation SL–SR (pelvis)	°	1.17 ± 5.47	0.97 (−1.96–4.42)	−10.08	12.1
Shoulder rotation SL–SR (baseline)	°	0.50 ± 2.60	0.54 (−0.76–1.83)	−5.13	5.53

SD, standard deviation; IQR, interquartile range; reference limits were defined as the 2.5th and 97.5th percentiles; ^‡^DIERS-derived parameters used to define the screening categories.

### 3.3 Group differences in DIERS-derived parameters

Differences in the 45 DIERS-derived parameters stratified by age group, sex, and screening category are presented in Table 5. The reported counts refer to the number of significant parameters, some of which may reflect the same postural construct and therefore should not be interpreted as independent findings. After FDR correction, 27 parameters showed statistically significant differences across age groups. Among these significant parameters, *η*^2^ values indicated trivial effects for 9 parameters, small effects for 15 parameters, and medium effects for 3 parameters; no large effects were observed. Overall, although age related differences were observed across multiple parameters, their effect sizes were generally small. Consistent with these findings, a sensitivity analysis treating age as a continuous variable and adjusting for sex showed a similar pattern, with age statistically associated with multiple parameters but generally explaining little additional variance (Supplementary Table S4).

**Table 5:** DIERS-derived parameters by age, sex, and screening category.

Parameter	Unit	18–39 (*n* = 628)	40–59 (*n* = 911)	≥60 (*n* = 320)	Age *η*^2^ (95% CI)	Male (*n* = 980)	Female (*n* = 879)	Sex Cohen's *d* (95% CI)	Scoliotic posture (*n* = 667)	Unclassified (*n* = 1085)	Scoliosis risk (*n* = 107)	Screening classification *η*^2^ (95% CI)
Distance measurements												
Trunk length VP–DM	mm	464.15 ± 33.29	465.06 ± 33.43	468.68 ± 34.09	0.00 (0.00–0.01)	483.53 ± 27.46	445.13 ± 27.53	1.40* (1.28–1.52)	466.02 ± 31.38	466.04 ± 34.42	454.61 ± 35.59	0.01* (0.00–0.02)
Trunk length VP–SP	mm	520.03 ± 33.01	518.84 ± 32.70	521.98 ± 33.71	0.00 (0.00–0.01)	536.85 ± 27.99	500.75 ± 27.21	1.31* (1.20–1.42)	519.77 ± 31.02	520.55 ± 33.85	512.03 ± 35.25	0.00 (0.00–0.01)
VP–SP/VP–DM	%	112.13 ± 2.60	111.67 ± 2.83	111.47 ± 2.82	0.01* (0.00–0.02)	111.08 ± 2.25	112.59 ± 3.05	0.57* (0.48–0.65)	111.62 ± 2.54	111.80 ± 2.77	112.77 ± 3.69	0.01* (0.00–0.02)
Dimple distance DL–DR	mm	95.43 ± 11.90	92.43 ± 13.98	89.48 ± 13.63	0.02* (0.01–0.04)	93.01 ± 12.36	92.86 ± 14.49	0.01 (0.00–0.11)	92.76 ± 11.55	93.35 ± 13.56	89.87 ± 20.51	0.00 (0.00–0.02)
DL–DR/ VP–DM	%	20.63 ± 2.78	19.98 ± 3.70	19.17 ± 3.12	0.02* (0.01–0.04)	19.28 ± 2.65	20.94 ± 3.81	0.51* (0.42–0.60)	19.97 ± 2.68	20.14 ± 3.53	19.88 ± 4.92	0.00 (0.00–0.01)
Trunk length T4–DM	mm	385.41 ± 27.39	386.17 ± 27.64	388.81 ± 28.30	0.00 (0.00–0.01)	401.45 ± 22.57	369.56 ± 22.72	1.41* (1.29–1.53)	386.97 ± 25.89	386.88 ± 28.45	377.46 ± 29.19	0.01* (0.00–0.02)
Trunk and pelvis imbalances												
Sagittal imbalance VP–DM	°	0.53 ± 2.39	0.78 ± 2.36	1.85 ± 2.50	0.03* (0.02–0.05)	1.17 ± 2.40	0.56 ± 2.44	0.25* (0.16–0.34)	0.79 ± 2.34	0.95 ± 2.48	0.79 ± 2.58	0.00 (0.00–0.01)
Sagittal imbalance VP–DM	mm	4.82 ± 19.48	6.73 ± 19.18	15.22 ± 20.42	0.03* (0.02–0.05)	10.15 ± 20.26	4.64 ± 18.91	0.28* (0.19–0.38)	6.79 ± 19.17	8.08 ± 20.15	6.81 ± 20.47	0.00 (0.00–0.01)
Coronal imbalance VP–DM	°	−0.63 ± 1.26	−0.53 ± 1.52	−0.45 ± 1.49	0.00 (0.00–0.01)	−0.56 ± 1.44	−0.55 ± 1.43	0.01 (0.00–0.09)	−0.47 ± 1.23	−0.64 ± 1.46	−0.16 ± 2.11	0.01* (0.00–0.02)
Coronal imbalance VP–DM	mm	−5.14 ± 10.13	−4.29 ± 12.18	−3.72 ± 12.38	0.00 (0.00–0.01)	−4.72 ± 12.08	−4.21 ± 10.96	0.04 (0.00–0.13)	−3.84 ± 9.83	−5.20 ± 11.75	−1.15 ± 17.49	0.01* (0.00–0.02)
Pelvic obliquity DL–DR	°	0.29 ± 4.40	0.33 ± 5.51	−0.19 ± 4.67	0.00 (0.00–0.01)	0.13 ± 4.32	0.33 ± 5.69	0.04 (0.00–0.12)	−0.03 ± 1.54	0.26 ± 5.28	1.42 ± 11.83	0.00 (0.00–0.03)
Pelvic obliquity DL–DR^‡^	mm	0.48 ± 6.91	0.64 ± 11.39	−0.30 ± 6.93	0.00 (0.00–0.01)	0.26 ± 6.85	0.60 ± 11.57	0.04 (0.00–0.13)	−0.03 ± 2.38	0.61 ± 9.83	1.35 ± 22.75	0.00 (0.00–0.02)
Pelvic torsion DL–DR	°	0.79 ± 2.78	0.45 ± 3.57	0.35 ± 3.06	0.00 (0.00–0.01)	0.76 ± 3.21	0.31 ± 3.26	0.14* (0.05–0.23)	0.52 ± 2.24	0.58 ± 3.02	0.45 ± 7.71	0.00 (0.00–0.02)
Pelvic inclination (dimples)	°	17.24 ± 5.55	16.17 ± 6.42	13.63 ± 6.91	0.04* (0.02–0.06)	14.07 ± 6.02	18.35 ± 5.93	0.72* (0.62–0.81)	16.57 ± 5.92	15.79 ± 6.50	16.20 ± 7.10	0.00 (0.00–0.01)
Pelvic inclination (symmetry line)	°	17.37 ± 7.29	16.43 ± 8.14	13.94 ± 8.23	0.02* (0.01–0.04)	12.62 ± 6.70	20.45 ± 7.19	1.13* (1.03–1.24)	16.71 ± 7.62	16.04 ± 8.14	16.73 ± 8.11	0.00 (0.00–0.01)
Pelvis rotation	°	−0.84 ± 3.28	−0.68 ± 3.36	−0.32 ± 3.60	0.00 (0.00–0.01)	−0.95 ± 3.27	−0.35 ± 3.47	0.18* (0.09–0.27)	−0.59 ± 2.85	−0.62 ± 3.42	−1.64 ± 5.34	0.01 (0.00–0.02)
Location of postural reference points												
ICT	mm	6.45 ± 10.21	6.11 ± 9.79	6.26 ± 8.81	0.00 (0.00–0.00)	4.76 ± 9.67	7.91 ± 9.62	0.33* (0.24–0.42)	5.87 ± 9.66	6.28 ± 9.79	8.35 ± 10.05	0.00 (0.00–0.01)
KA	mm	−159.56 ± 45.81	−165.29 ± 42.24	−177.65 ± 39.74	0.02* (0.01–0.03)	−183.91 ± 35.66	−144.94 ± 42.22	1.00* (0.91–1.11)	−164.24 ± 44.01	−166.45 ± 43.23	−163.41 ± 42.85	0.00 (0.00–0.01)
ITL	mm	−307.02 ± 40.36	−313.09 ± 39.79	−319.97 ± 43.12	0.01* (0.00–0.02)	−330.33 ± 37.30	−292.03 ± 34.59	1.06* (0.96–1.17)	−310.75 ± 40.25	−313.32 ± 41.47	−310.29 ± 37.06	0.00 (0.00–0.01)
LA	mm	−390.31 ± 39.76	−397.72 ± 40.98	−403.81 ± 45.74	0.01* (0.01–0.03)	−420.05 ± 34.38	−369.75 ± 32.01	1.51* (1.39–1.63)	−395.38 ± 39.96	−397.56 ± 43.16	−388.72 ± 36.01	0.00 (0.00–0.01)
ILS	mm	−470.05 ± 39.30	−471.86 ± 38.47	−470.91 ± 41.18	0.00 (0.00–0.01)	−494.34 ± 29.78	−445.16 ± 31.45	1.61* (1.49–1.74)	−471.85 ± 37.35	−471.59 ± 40.51	−461.21 ± 35.99	0.00* (0.00–0.01)
Flèche cervicale	mm	55.91 ± 18.12	60.87 ± 18.44	71.20 ± 21.65	0.07* (0.05–0.10)	68.91 ± 18.70	52.12 ± 16.59	0.95* (0.86–1.04)	59.55 ± 19.23	61.64 ± 19.86	63.13 ± 18.92	0.00 (0.00–0.01)
Flèche lombaire	mm	40.31 ± 12.51	41.25 ± 12.52	40.67 ± 12.16	0.00 (0.00–0.01)	41.22 ± 12.55	40.40 ± 12.35	0.07 (0.00–0.16)	40.73 ± 12.22	40.80 ± 12.60	41.78 ± 12.57	0.00 (0.00–0.00)
Spinal curve angles												
Kyphotic angle ICT–ITL	°	47.41 ± 8.68	50.40 ± 8.05	54.94 ± 9.47	0.08* (0.06–0.11)	50.86 ± 8.58	49.41 ± 9.17	0.16* (0.07–0.26)	49.48 ± 8.39	50.25 ± 9.05	53.68 ± 9.48	0.01* (0.00–0.02)
Kyphotic angle VP–ITL	°	43.84 ± 8.25	46.53 ± 8.08	50.82 ± 9.29	0.07* (0.05–0.10)	47.49 ± 8.52	45.09 ± 8.69	0.28* (0.18–0.37)	45.87 ± 8.17	46.41 ± 8.92	48.84 ± 8.97	0.01* (0.00–0.02)
Kyphotic angle VP–T12	°	40.64 ± 7.60	42.78 ± 7.45	45.81 ± 8.11	0.05* (0.03–0.07)	43.32 ± 7.69	41.75 ± 7.88	0.20* (0.11–0.29)	42.44 ± 7.47	42.51 ± 7.99	44.11 ± 8.07	0.00 (0.00–0.01)
Lordotic angle ITL–ILS	°	36.74 ± 8.72	36.67 ± 8.81	35.44 ± 8.60	0.00 (0.00–0.01)	33.29 ± 7.55	40.05 ± 8.63	0.84* (0.74–0.93)	36.29 ± 8.42	36.30 ± 8.77	39.59 ± 10.01	0.01* (0.00–0.02)
Lordotic angle ITL–DM	°	33.00 ± 8.78	32.50 ± 8.82	30.75 ± 8.17	0.01* (0.00–0.02)	28.82 ± 7.42	36.32 ± 8.38	0.95* (0.86–1.04)	32.47 ± 8.42	32.10 ± 8.83	34.42 ± 9.42	0.00 (0.00–0.01)
Lordotic angle T12–DM	°	29.80 ± 8.85	28.75 ± 9.18	25.74 ± 8.69	0.02* (0.01–0.04)	24.64 ± 7.67	32.98 ± 8.51	1.03* (0.93–1.13)	29.03 ± 8.64	28.20 ± 9.28	29.68 ± 9.68	0.00 (0.00–0.01)
Spinal deviation												
Vertebral rotation (RMS) ^‡^	°	3.56 ± 1.66	3.71 ± 1.70	3.89 ± 1.78	0.00* (0.00–0.01)	3.64 ± 1.65	3.75 ± 1.76	0.07 (0.00–0.16)	2.96 ± 0.97	3.80 ± 1.50	7.14 ± 2.52	0.31* (0.27–0.35)
Vertebral rotation (max)	°	0.55 ± 7.72	0.23 ± 7.97	0.92 ± 8.61	0.00 (0.00–0.01)	0.03 ± 7.78	0.92 ± 8.22	0.11* (0.03–0.20)	0.68 ± 6.76	0.52 ± 8.11	−1.55 ± 12.57	0.00 (0.00–0.02)
Vertebral rotation (+max)	°	4.70 ± 4.05	4.77 ± 4.06	5.72 ± 4.36	0.01* (0.00–0.02)	4.59 ± 3.97	5.27 ± 4.26	0.16* (0.08–0.25)	4.40 ± 3.42	5.08 ± 4.15	6.44 ± 6.66	0.01* (0.00–0.03)
Vertebral rotation (−max)	°	−4.53 ± 3.17	−5.01 ± 3.21	−5.17 ± 3.22	0.01* (0.00–0.02)	−4.98 ± 3.13	−4.76 ± 3.28	0.07 (0.00–0.15)	−4.07 ± 2.52	−5.08 ± 3.06	−7.92 ± 5.55	0.08* (0.05–0.12)
Vertebral rotation (amplitude)	°	9.23 ± 4.04	9.79 ± 4.06	10.89 ± 4.19	0.02* (0.01–0.03)	9.58 ± 4.12	10.03 ± 4.10	0.11* (0.02–0.20)	8.47 ± 3.33	10.15 ± 4.03	14.36 ± 5.25	0.11* (0.09–0.15)
Trunk torsion	°	1.16 ± 4.61	1.09 ± 5.50	1.33 ± 6.71	0.00 (0.00–0.01)	0.96 ± 5.44	1.37 ± 5.46	0.08 (0.00–0.17)	1.15 ± 4.73	1.23 ± 5.61	0.36 ± 7.56	0.00 (0.00–0.01)
Apical deviation VP–DM (RMS) ^‡^	mm	4.60 ± 2.62	4.86 ± 2.81	5.61 ± 3.27	0.01* (0.01–0.03)	5.07 ± 3.04	4.72 ± 2.61	0.12* (0.03–0.22)	3.25 ± 1.01	5.48 ± 2.71	9.31 ± 4.61	0.28* (0.25–0.32)
Apical deviation VP–DM (max)	mm	2.86 ± 9.05	4.21 ± 8.90	4.62 ± 10.74	0.01* (0.00–0.02)	4.19 ± 9.56	3.41 ± 9.02	0.08 (0.00–0.17)	1.98 ± 6.25	4.58 ± 9.72	7.59 ± 16.37	0.03* (0.01–0.05)
Apical deviation VP–DM (+max)	mm	6.51 ± 5.08	7.28 ± 5.16	8.44 ± 5.98	0.02* (0.01–0.03)	7.48 ± 5.62	6.93 ± 4.96	0.10* (0.01–0.19)	5.02 ± 2.61	8.03 ± 5.28	12.71 ± 10.05	0.14* (0.11–0.17)
Apical deviation VP–DM (−max)	mm	−4.20 ± 3.72	−3.80 ± 3.53	−4.49 ± 4.71	0.00* (0.00–0.01)	−4.03 ± 3.93	−4.08 ± 3.72	0.01 (0.00–0.11)	−3.66 ± 2.49	−4.12 ± 4.11	−5.80 ± 6.43	0.02* (0.00–0.04)
Apical deviation VP–DM (amplitude)	mm	10.71 ± 4.91	11.08 ± 4.81	12.93 ± 5.97	0.02* (0.01–0.04)	11.51 ± 5.41	11.01 ± 4.76	0.10* (0.01–0.19)	8.68 ± 2.66	12.15 ± 4.92	18.51 ± 8.01	0.22* (0.19–0.26)
Shoulder parameters												
Shoulder distance SL–SR	mm	308.88 ± 24.76	307.75 ± 23.07	305.03 ± 21.08	0.00 (0.00–0.01)	321.03 ± 18.75	292.76 ± 18.46	1.52* (1.40–1.64)	306.89 ± 22.59	308.63 ± 23.87	302.71 ± 22.17	0.00* (0.00–0.01)
Shoulder tilt SL–SR	°	−0.12 ± 1.45	−0.25 ± 1.44	−0.07 ± 1.49	0.00 (0.00–0.01)	−0.15 ± 1.45	−0.20 ± 1.45	0.03 (0.00–0.12)	−0.31 ± 1.35	−0.08 ± 1.49	−0.21 ± 1.63	0.01* (0.00–0.02)
Shoulder tilt SL–SR	mm	−0.67 ± 7.79	−1.35 ± 7.79	−0.35 ± 7.89	0.00 (0.00–0.01)	−0.88 ± 8.14	−1.03 ± 7.43	0.02 (0.00–0.11)	−1.74 ± 7.22	−0.45 ± 8.04	−1.14 ± 8.60	0.01* (0.00–0.02)
Shoulder rotation SL–SR (pelvis)	°	1.39 ± 5.39	1.29 ± 5.44	0.41 ± 5.68	0.00* (0.00–0.01)	1.55 ± 5.16	0.75 ± 5.78	0.15* (0.06–0.24)	1.05 ± 4.63	1.14 ± 5.37	2.32 ± 9.68	0.00 (0.00–0.02)
Shoulder rotation SL–SR (baseline)	°	0.55 ± 2.53	0.61 ± 2.62	0.08 ± 2.65	0.01* (0.00–0.01)	0.60 ± 2.34	0.39 ± 2.86	0.08 (0.00–0.18)	0.46 ± 2.28	0.51 ± 2.48	0.69 ± 4.79	0.00 (0.00–0.01)

‡ DIERS derived parameters used to define the screening categories. Comparisons across age groups and screening categories were performed using Welch one way analysis of variance and effect sizes were reported as eta squared (*η*^2^) with 95% confidence intervals. Sex comparisons were performed using Welch independent samples *t*-tests and effect sizes were reported as the absolute value of Cohen's *d* with 95% confidence intervals. *P* values were adjusted using the Benjamini Hochberg false discovery rate procedure separately within the 45 comparisons for age, sex, and screening category. Asterisks indicate FDR adjusted *P* values: *P < 0.05.

In the sex-stratified comparisons, 31 parameters showed statistically significant differences. Among these significant parameters, Cohen's *d* indicated negligible effects for 10 parameters, small effects for 5 parameters, medium effects for 3 parameters, and large effects for 13 parameters. Medium effects were mainly observed in the distance measurements and trunk and pelvis imbalance domains, whereas large sex-related differences were concentrated in distance measurements, locations of postural reference points, spinal curve angles, and shoulder parameters.

Across screening categories, 21 parameters showed statistically significant between-group differences. After excluding the two statistically significant parameters used to define the screening categories, 19 significant parameters remained. Only four parameters showed medium-to-large effects. These four parameters represented related measures of the apical deviation and vertebral rotation constructs and were moderately to strongly correlated with the corresponding screening parameters (|*r*| = 0.42–0.83; Supplementary Figure S1). The other 15 significant parameters showed only trivial or small effects, including 11 with trivial effects and 4 with small effects.

### 3.4 Dimensional structure of DIERS-derived parameters

Unique variable analysis (UVA) was used to remove redundant DIERS-derived parameters before network analysis. This procedure removed 21 parameters and retained 24 non-redundant parameters for EGA (Supplementary Table S5). Among the retained parameters, only two pairs represented alternative statistical summaries of the same parameter construct: apical deviation VP-DM (RMS) and apical deviation VP-DM (max), and vertebral rotation (RMS) and vertebral rotation (max). EGA of the retained parameters identified a five-dimension solution. Bootstrap EGA supported this solution in 98.5% of the 1,000 bootstrap samples, indicating a stable clustering structure among the retained parameters. Figure 1 shows the EGA network and parameter clustering across the five dimensions. Table 6 reports the standardized network loadings (SNL) for each retained parameter. Larger absolute SNL values indicate stronger representation of the assigned dimension. The sign of the SNL indicates the loading direction within that dimension.

**Figure 1:**
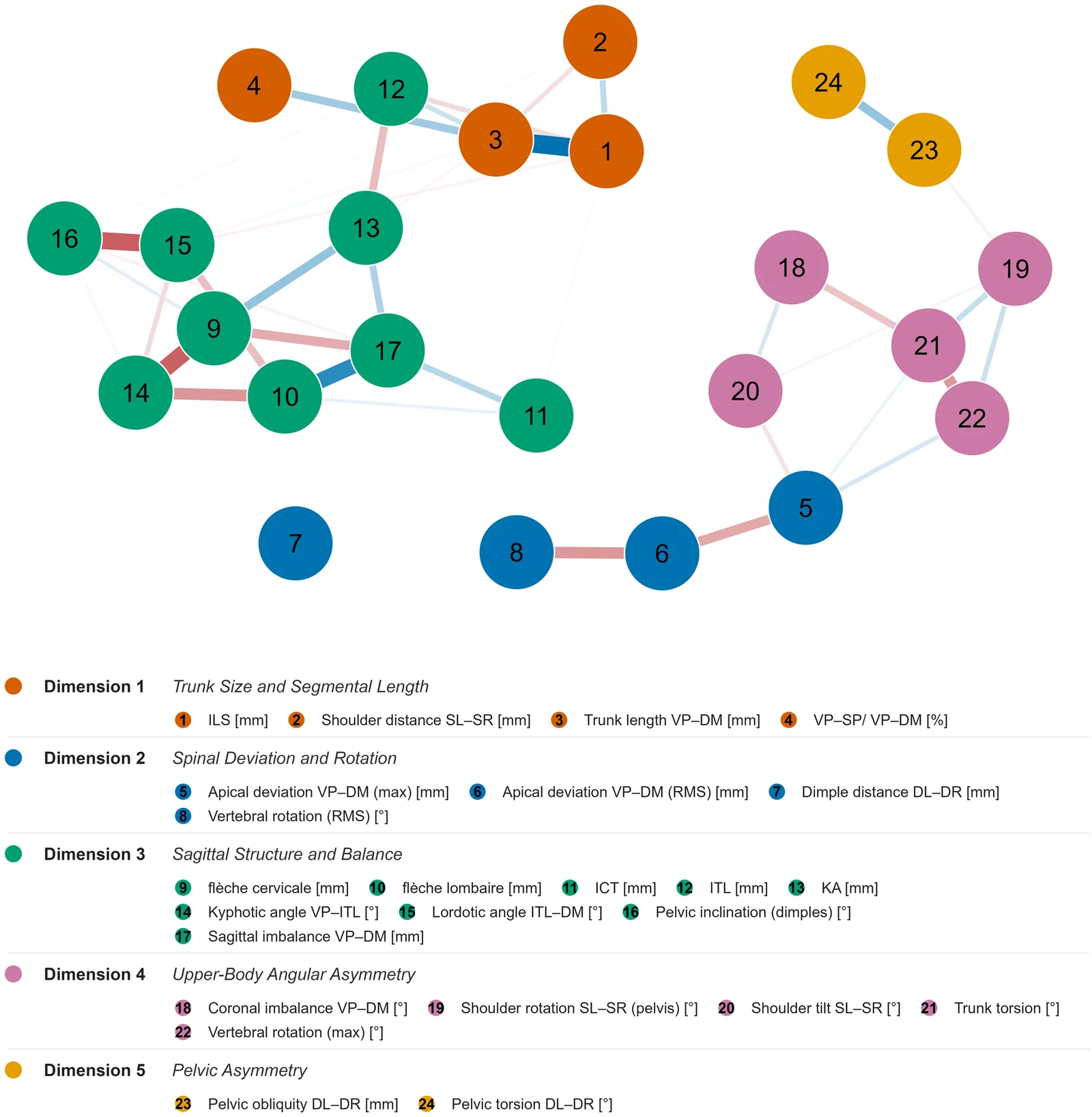
EGA network of DIERS-derived parameters. Nodes represent the 24 parameters retained after UVA and are colored according to their EGA dimension. Node numbers correspond to the parameter labels listed below the network. Descriptive labels for the five dimensions are provided alongside each dimension. Edge color indicates association direction (red = positive, blue = negative), and edge thickness reflects association strength, with thicker edges indicating stronger associations..

**Table 6:** EGA standardized network loadings.

DIERS-derived parameter	Unit	Dim 1	Dim 2	Dim 3	Dim 4	Dim 5
Trunk length VP–DM	mm	**0** **.** **77**	−0.04	−0.19	0.00	0.00
ILS	mm	**−0**.**61**	−0.00	0.18	−0.00	−0.00
Shoulder distance SL–SR	mm	**0**.**25**	0.00	−0.06	0.00	0.00
VP–SP/VP–DM	%	**−0**.**19**	0.01	−0.01	−0.00	−0.00
Apical deviation VP–DM (RMS)	mm	0.00	**0**.**68**	−0.00	0.00	0.00
Vertebral rotation (RMS)	°	−0.03	**0**.**38**	0.01	0.00	0.00
Apical deviation VP–DM	mm	0.00	**0**.**31**	0.00	−0.23	0.00
Dimple distance DL–DR	mm	0.00	**−0**.**01**	−0.00	0.00	−0.00
flèche cervicale	mm	0.03	−0.00	**−0**.**80**	−0.00	−0.00
Sagittal imbalance VP–DM	mm	−0.00	−0.00	**−0**.**71**	−0.00	−0.00
KA	mm	−0.07	0.00	**0**.**48**	0.00	0.00
Lordotic angle ITL–DM	°	−0.11	0.00	**0**.**42**	0.00	0.00
Pelvic inclination (dimples)	°	0.00	−0.00	**0**.**39**	0.00	0.00
ITL	mm	−0.21	0.00	**0**.**20**	0.00	0.00
Flèche lombaire	mm	0.04	0.00	**0**.**12**	0.00	0.00
ICT	mm	−0.06	0.02	**0**.**04**	0.01	0.00
Kyphotic angle VP–ITL	°	−0.00	0.01	**−0**.**03**	−0.00	−0.00
Trunk torsion	°	0.00	−0.07	0.00	**0**.**61**	0.00
Vertebral rotation (max)	°	0.00	−0.10	0.01	**0**.**44**	0.00
Shoulder rotation SL–SR (pelvis)	°	−0.00	−0.00	−0.00	**−0**.**32**	−0.08
Coronal imbalance VP–DM	°	0.00	0.00	0.00	**0**.**31**	−0.01
Shoulder tilt SL–SR	°	−0.00	0.10	−0.00	**−0**.**17**	−0.00
Pelvic torsion DL–DR	°	0.00	0.00	0.00	−0.01	**0**.**52**
Pelvic obliquity DL–DR	mm	−0.00	−0.00	−0.00	−0.06	**−0**.**52**

Dim, dimension identified by EGA. Larger absolute standardized network loading values indicate stronger representation of the corresponding dimension, and the sign indicates loading direction. Bold values indicate each parameter's assigned EGA dimension.

Dimension 1 comprised four parameters and was mainly characterized by trunk length and related anatomical distance measures. Trunk length VP-DM showed the largest loading (SNL = 0.77). ILS also showed a high absolute loading (SNL = −0.61). This dimension was therefore labelled Trunk Size and Segmental Length.

Dimension 2 comprised four parameters related mainly to coronal spinal deviation and axial vertebral rotation. Apical deviation VP–DM (RMS) and apical deviation VP–DM (max), representing two statistical summaries of the same apical-deviation construct, were both assigned to this dimension. Apical deviation VP–DM (RMS) showed the largest loading (SNL = 0.68), followed by vertebral rotation (RMS) (SNL = 0.38). These two parameters corresponded to the screening indicators used in the categorical analyses. Dimple distance DL–DR had an SNL close to zero, indicating a weak contribution to this dimension. This dimension was therefore labelled Spinal Deviation and Rotation.

Dimension 3 was the largest cluster and included nine parameters. The largest absolute loadings were observed for flèche cervicale (SNL = −0.80) and sagittal imbalance VP-DM (SNL = −0.71). Parameters in this dimension were mainly derived from postural reference point locations and spinal curve angles. This dimension was therefore labelled Sagittal Structure and Balance.

Dimension 4 comprised five parameters, all of which were angular measurements related to the trunk, spine, and shoulders. Trunk torsion showed the largest loading (SNL = 0.61), followed by vertebral rotation (max) (SNL = 0.44). Shoulder rotation SL-SR (pelvis) loaded in the opposite direction (SNL = −0.32). This dimension was therefore labelled Upper-Body Angular Asymmetry.

Dimension 5 comprised two pelvic asymmetry-related parameters: pelvic torsion DL-DR and pelvic obliquity DL-DR. These parameters showed loadings in opposite directions, with SNL values of 0.52 and −0.52, respectively. This dimension was therefore labelled Pelvic Asymmetry.

## 4 Discussion

This study characterized 45 upper body posture parameters derived from DIERS surface topography in 1,859 adults and examined how these parameters varied by sex, age, and screening category. Sex-related differences were substantial, whereas age-related differences were predominantly small and screening-category differences were generally limited, with the more pronounced effects mainly confined to parameters related to the classification criteria. EGA further identified five stable dimensions among the retained parameters, revealing a structured multidimensional organization within the parameter set.

Directly comparable parameters from the present study were compared with two previously published adult reference datasets (Supplementary Tables S2, S3, respectively). The degree of agreement varied across parameters. Relatively similar values were observed for some trunk length measures and some kyphotic angles, whereas larger differences were observed for sagittal imbalance and several spinal deviation measures. Given the differences in study populations and measurement systems, some systematic between-study variation would be expected. These comparisons are therefore provided as contextual information for the reference values reported in the present study.

In the present study, the mean adult Kyphotic angle ICT–ITL was 50.17, closely matching the value of 50.03 reported by Kaps et al. in 201 adults (34), suggesting that the level of thoracic kyphosis in our sample was broadly consistent with previous adult reference data. Age-group analysis further showed that both Kyphotic angle ICT–ITL and Kyphotic angle VP–ITL increased with age, with medium effect sizes, consistent with a previous systematic review reporting an age-related increase in kyphotic angle (35). Flèche cervicale showed a similar age-related pattern. The mean Flèche cervicale value in the present study was 60.97, close to values reported in two previous large-sample studies of 1,149 and 1,127 participants, which reported mean values of 64.80 (36) and 64.25 (37), respectively. In our age-group analysis, Flèche cervicale also increased with age and showed a medium effect size (36). Because Flèche cervicale is defined as the horizontal distance from the cervical flèche point to the vertical reference line passing through KA, increased thoracic kyphosis may enlarge this distance by altering thoracic sagittal morphology (38, 39). The moderate correlation between Flèche cervicale and Kyphotic angle VP–ITL in the present study (*r* = 0.67; Supplementary Appendix 1) further supports this interpretation. By contrast, the remaining 24 parameters that differed between age groups showed mainly small or trivial effect sizes, suggesting that the practical relevance of these age-related differences may be limited. Because the DIERS system estimates these parameters using a surface topography-based modelling algorithm, they may be affected by skin, subcutaneous fat, and soft-tissue distribution (40). Therefore, these small-effect differences should be interpreted cautiously, as they may partly reflect true postural changes but may also be influenced by measurement error or individual variation in surface morphology.

Sex-stratified analysis showed statistically significant differences between male and female participants in 31 DIERS-derived parameters, 13 of which had large effect sizes. These findings indicate pronounced sex-related differences in the distribution of adult DIERS-derived parameters. Previous raster-stereographic studies have shown that men are generally taller and heavier than women and that these anthropometric differences are reflected in size-related parameters such as trunk length and scapular distance (22, 41). Therefore, some of the large sex-related differences observed in the present study, particularly in distance measurements, may partly reflect anthropometric differences between men and women. Sex stratification is therefore recommended when establishing reference ranges, although the contribution of anthropometric differences remains uncertain. Future studies should include height, weight, and BMI and examine whether sex-related differences remain after adjustment for these factors.

Screening categories were defined using three measures: pelvic obliquity DL–DR (mm), vertebral rotation (RMS), and apical deviation VP–DM (RMS). Using these criteria, 107 participants (5.8%) were assigned to the scoliosis risk group. Twenty-one differed significantly between screening groups. Among these, four parameters belonged to the Spinal Deviation domain, showed medium-to-large between-group effect sizes, and were moderately to strongly correlated with the parameters used to define the screening categories. Because scoliosis is clinically characterized by lateral curvature in the coronal plane accompanied by axial rotation, these pronounced differences in spinal deviation parameters were expected. Outside the constructs used to define the screening categories and their related representations, the remaining between-group differences were limited to trivial or small effect sizes. A recent large-sample radiographic study comparing 1,779 individuals with adolescent idiopathic scoliosis and 1,115 normal adolescents and young adults showed significant differences in sagittal spinal alignment (42). However, our findings showed relatively limited differences in sagittal-plane parameters. Kyphotic angle ICT–ITL and Kyphotic angle VP–ITL reached statistical significance, but with small effect sizes, whereas Lordotic angle ITL–ILS, Lordotic angle ITL–DM, and Lordotic angle T12–DM showed no significant between-group differences. This pattern may partly reflect the screening classification used in the present study, which was defined using DIERS-derived parameters primarily representing coronal and axial alterations. This emphasis is also broadly consistent with conventional scoliosis screening, which relies mainly on trunk asymmetry and axial rotation, whereas radiographic diagnosis is based on coronal curvature. Nevertheless, limited between-group differentiation does not imply limited clinical relevance of sagittal-plane parameters (43, 44). Previous biomechanical modelling has shown that alterations in thoracic and lumbar curvature can influence thoracolumbar loading in the context of spinal deformity (45). In healthy populations, physiological variation in kyphosis and lordosis has also been shown to modulate spinal loading patterns (46). Thus, sagittal-plane parameters may provide limited differentiation within the present screening classification while remaining biomechanically relevant.

EGA is a network psychometric dimensionality reduction method that identifies latent dimensional structure in data by estimating partial correlations among variables (32). Most redundant representations of the same parameter construct were removed by UVA before EGA. Limited construction-related similarity remained among the retained parameters. EGA identified five dimensions suggesting an organization of upper body posture parameters beyond their original measurement-domain classification. Dimension 1 (Trunk Size and Segmental Length) captures the overall size and segmental proportions of the trunk. Dimension 2 (Coronal Deviation and Axial Rotation) clusters parameters used for scoliosis risk categories and may therefore be closely associated with scoliosis risk. Dimension 3 (Sagittal Spinal Structure and Postural Balance) suggests that sagittal spinal structure may function as a multisegmented coordinated system, in which thoracic curvature, lumbar curvature, and overall postural balance in the sagittal plane may be interrelated, rather than each spinal segment changing independently. Dimension 4 (Upper Body Angular Asymmetry) groups parameters reflecting rotational changes in the upper body into a distinct dimension. Dimension 5 (Pelvic Asymmetry) includes only two parameters (pelvic torsion DL–DR and pelvic obliquity DL–DR), forming an independent pelvic-level assessment dimension. These findings suggest that upper body postural parameters may exhibit coordinated patterns of variation rather than changing independently across individual structures (47, 48). Importantly, these patterns were not limited to alternative statistical representations of the same parameter, as measures reflecting different constructs were also grouped within the same dimension. This dimensional structure may therefore inform the clinical interpretation of parameter thresholds, suggesting that an individual parameter should not be interpreted in isolation but alongside other parameters within the same dimension. However, the clinical validity of this dimension-based interpretation requires further evaluation against appropriate gold standard measures.

### 4.1 Technical considerations

From a technical perspective, advances in low-dose radiographic imaging have substantially reduced radiation exposure during spinal assessment, and radiography remains an important tool for the diagnosis of scoliosis and the quantification of spinal structure (18). In contrast, raster stereography estimates spinal structure from the surface morphology of the back. Its measurements may therefore be influenced by factors such as BMI, tattoos, and postural fluctuations, which can introduce errors into the estimation of spinal structure (13). However, scoliosis often requires repeated assessment and long-term follow-up, and the absence of ionizing radiation makes raster stereography more feasible for screening and frequent monitoring as a complement to radiography (20). In a screening pathway, individuals with raster-stereographic findings suggestive of scoliosis could subsequently undergo radiographic assessment for diagnostic confirmation and structural quantification. In this context, the reference values established in the present study for East Asian adults provide population-based benchmarks for interpreting raster-stereographic screening results. In the future, as larger population datasets become available, machine-learning approaches may help further improve the accuracy of spinal structure estimation (49, 50).

### 4.2 Limitations

This study has several limitations. First, it employed a cross-sectional design with a sample drawn from a single-center health check-up population, which may not represent the broader adult population. Second, surface topography measurements are influenced by soft-tissue thickness, subcutaneous fat distribution, and body habits. However, anthropometric data such as height and weight were not available in the present dataset, precluding assessment of their potential confounding effects on the parameter distributions. Third, the scoliosis screening categories reported here were defined using DIERS-derived parameters without independent validation against radiographic Cobb angle. These categories should therefore be interpreted as screening signals rather than diagnostic evidence.

## 5 Conclusions

This study established population reference ranges for 45 DIERS-derived upper body posture parameters in 1,859 adults, providing an evidence base for future spinal health management. Sex-related differences were pronounced, with 31 of 45 parameters showing statistically significant differences, including 13 with large effects, suggesting the use of sex-stratified reference values in clinical interpretation. By comparison, age-related effects were predominantly trivial to small. Five stable dimensions were identified in this study, suggesting that changes in upper body posture may follow specific patterns that span measurement domains. These findings indicate that future research should consider multi-structural coordination and specific patterns of spinal morphological change.

## Data Availability

The raw data supporting the conclusions of this article will be made available by the authors, without undue reservation.
