# Supplementary material for: Reference ranges and dimensional structure of upper body posture parameters in adults: a cross-sectional study
Source: Front Rehabil Sci. 2026 Sep 30;7:1930857. doi: 10.3389/fresc.2026.1930857 (PMC13662147; doi:10.3389/fresc.2026.1930857)
Supplement: Supplementary file 1 [file Datasheet1.docx]

Figure S1 Spearman correlation matrix of 45 spinal function parameters. Parameters are grouped into six anatomical domains: Length Measurements, Trunk and Pelvis Imbalances, Location of Postural Reference Points, Spinal Curve Angles, Spinal Deviation, and Shoulder-related Parameters. Thick black lines demarcate domain boundaries, with domain labels annotated above and to the right of the heatmap. Color intensity reflects correlation strength (blue = −1, white = 0, red = +1). The color bar at the bottom provides the reference scale for Spearman's ρ.

Figure S2 Weighted topological overlap (wTO) matrix of the 45 spinal function parameters, derived from the GLASSO network estimated during Unique Variable Analysis. wTO quantifies the similarity of topological positions occupied by two parameters in the network. Values range from 0 (white; completely distinct connectivity profiles) to 1 (dark red; identical connectivity profiles).

Table S1. Age Trends in Screening Categories

| **Screening category** | **18–39 years, n/N (%)** | **40–59 years, n/N (%)** | **≥60 years, n/N (%)** | **P** | **Adjusted P** |
| --- | --- | --- | --- | --- | --- |
| Scoliotic posture | 238/628 (37.9) | 326/911 (35.8) | 103/320 (32.2) | 0.088 | 0.132 |
| Scoliosis risk | 27/628 (4.3) | 54/911 (5.9) | 26/320 (8.1) | 0.016 | 0.049 |
| Unclassified | 363/628 (57.8) | 531/911 (58.3) | 191/320 (59.7) | 0.599 | 0.599 |
| Note. Trends across ordered age groups were examined using the Cochran–Armitage trend test. Adjusted P values were corrected using the Benjamini–Hochberg false discovery rate procedure across the three screening categories. A two-sided P < 0.05 was considered statistically significant. | | | | | |

| **Table S2. Overall-sample comparison with previously published adult reference data** | | | |
| --- | --- | --- | --- |
| **Parameter** | **Unit** | **^a^N=1,859, Mean ± SD** | **^b^N=201, Mean ± SD** |
| Sagittal imbalance VP–DM | ° | 0.88 ± 2.43 | 3.1 ± 2.1 |
| Sagittal imbalance VP–DM | mm | 7.54 ± 19.82 | 26.0 ± 17.5 |
| Coronal imbalance VP–DM | ° | −0.55 ± 1.43 | −0.2 ± 0.8 |
| Coronal imbalance VP–DM | mm | −4.48 ± 11.57 | −1.7 ± 7.3 |
| Kyphotic angle ICT–ITL | ° | 50.17 ± 8.89 | 49.9 ± 8.3 |
| Lordotic angle ITL–ILS | ° | 36.49 ± 8.75 | 40.9 ± 9.2 |
| Vertebral rotation (RMS) | ° | 3.69 ± 1.70 | 2.3 ± 0.9 |
| Vertebral rotation (max) | ° | 0.46 ± 8.00 | −1.9 ± 4.0 |
| Vertebral rotation (+max) | ° | 4.91 ± 4.12 | 1.5 ± 1.4 |
| Vertebral rotation (−max) | ° | −4.88 ± 3.20 | −3.3 ± 2.3 |
| Apical deviation VP–DM (RMS) | mm | 4.90 ± 2.85 | 3.6 ± 1.7 |
| Apical deviation VP–DM (max) | mm | 3.82 ± 9.31 | −1.8 ± 7.1 |
| Apical deviation VP–DM (+max) | mm | 7.22 ± 5.32 | 3.2 ± 3.0 |
| Apical deviation VP–DM (−max) | mm | −4.05 ± 3.83 | −4.9 ± 3.3 |
| Note: ᵃ Present study; ᵇ Huthwelker et al. (2022); | | | |

| **Table S3. Sex-specific comparison with previously published adult reference data** | | | | | | | |
| --- | --- | --- | --- | --- | --- | --- | --- |
| **Parameter** | **Unit** | **^a^Male (n=980), Mean ± SD** | **^a^Female (n=879), Mean ± SD** | **^b^Male (n=69), Mean ± SD** | **^b^Female (n=132), MoM ± SD** | **^c^Male (n=393), Median [P2.5–P97.5]** | **^c^Female (n=407), Median [P2.5–P97.5]** |
| Trunk length VP–DM | mm | 483.53 ± 27.46 | 445.13 ± 27.53 | — | — | 488.76 [424.15–544.42] | 453.51 [404.85–503.54] |
| Dimple distance DL–DR | mm | 93.01 ± 12.36 | 92.86 ± 14.49 | — | — | 98.11 [70.54–148.54] | 97.96 [71.86–128.28] |
| Sagittal imbalance VP–DM | ° | 1.17 ± 2.40 | 0.56 ± 2.44 | 3.0 ± 2.0 | 3.1 ± 2.1 | −3.24 [−8.38–1.85] | −3.29 [−10.38–1.80] |
| Sagittal imbalance VP–DM | mm | 10.15 ± 20.26 | 4.64 ± 18.91 | 26.7 ± 17.4 | 25.6 ± 17.6 | — | — |
| Coronal imbalance VP–DM | ° | −0.56 ± 1.44 | −0.55 ± 1.43 | −0.2 ± 0.8 | −0.2 ± 0.9 | −0.16 [−3.03–2.33] | −0.17 [−2.56–2.34] |
| Coronal imbalance VP–DM | mm | −4.72 ± 12.08 | −4.21 ± 10.96 | −2.0 ± 6.6 | −1.6 ± 7.7 | — | — |
| Pelvic obliquity DL–DR | ° | 0.13 ± 4.32 | 0.33 ± 5.69 | — | — | −0.42 [−4.26–3.81] | −0.42 [−3.93–4.52] |
| Pelvic obliquity DL–DR | mm | 0.26 ± 6.85 | 0.60 ± 11.57 | — | — | −0.72 [−7.40–6.50] | −0.72 [−7.47–8.43] |
| Pelvic torsion DL–DR | ° | 0.76 ± 3.21 | 0.31 ± 3.26 | — | — | 0.46 [−11.17–11.78] | 0.30 [−8.53–12.63] |
| Pelvis rotation | ° | −0.95 ± 3.27 | −0.35 ± 3.47 | — | — | −0.16 [−7.50–5.87] | 1.18 [−5.68–7.33] |
| Kyphotic angle ICT–ITL | ° | 50.86 ± 8.58 | 49.41 ± 9.17 | 49.6 ± 8.6 | 50.1 ± 8.2 | 51.29 [31.55–70.13] | 56.28 [31.85–92.48] |
| Lordotic angle ITL–ILS | ° | 33.29 ± 7.55 | 40.05 ± 8.63 | 34.9 ± 7.3 | 44.0 ± 8.5 | — | — |
| Lordotic angle ITL–DM | ° | 28.82 ± 7.42 | 36.32 ± 8.38 | — | — | 31.96 [14.89–48.71] | 49.02 [23.80–85.57] |
| Vertebral rotation (RMS) | ° | 3.64 ± 1.65 | 3.75 ± 1.76 | 2.3 ± 0.9 | 2.2 ± 0.9 | 3.54 [1.44–10.26] | 4.18 [1.53–10.31] |
| Vertebral rotation (max) | ° | 0.03 ± 7.78 | 0.92 ± 8.22 | −2.1 ± 4.0 | −1.8 ± 4.0 | — | — |
| Vertebral rotation (+max) | ° | 4.59 ± 3.97 | 5.27 ± 4.26 | 1.7 ± 1.4 | 1.5 ± 1.4 | — | — |
| Vertebral rotation (−max) | ° | −4.98 ± 3.13 | −4.76 ± 3.28 | −3.6 ± 2.3 | −3.2 ± 2.3 | — | — |
| Apical deviation VP–DM (RMS) | mm | 5.07 ± 3.04 | 4.72 ± 2.61 | 3.8 ± 1.6 | 3.5 ± 1.7 | 3.77 [1.30–9.60] | 3.84 [1.31–9.54] |
| Apical deviation VP–DM (max) | mm | 4.19 ± 9.56 | 3.41 ± 9.02 | −0.8 ± 7.7 | −2.3 ± 6.7 | — | — |
| Apical deviation VP–DM (+max) | mm | 7.48 ± 5.62 | 6.93 ± 4.96 | 3.8 ± 3.4 | 2.9 ± 2.8 | — | — |
| Apical deviation VP–DM (−max) | mm | −4.03 ± 3.93 | −4.08 ± 3.72 | −4.7 ± 3.2 | −4.9 ± 3.3 | — | — |
| Note: ᵃ Present study; ᵇ Huthwelker et al. (2022); ᶜ Ohlendorf et al. (2023). | | | | | | | |

| **Supplementary Table S4. Continuous associations between age and DIERS-derived parameters** | | | |
| --- | --- | --- | --- |
| **Parameter** | **F** | **FDR adjusted P** | **ΔR² for age, %** |
| Trunk length VP–DM | 3.17 | .019 | 0.62 |
| Trunk length VP–SP | 2.29 | .076 | 0.49 |
| VP–SP/VP–DM | 6.08 | <.001 | 0.95 |
| Dimple distance DL–DR | 12.25 | <.001 | 2.66 |
| DL–DR/VP–DM | 10.50 | <.001 | 2.03 |
| Trunk length T4–DM | 3.25 | .017 | 0.66 |
| Sagittal imbalance VP–DM (°) | 19.07 | <.001 | 4.36 |
| Sagittal imbalance VP–DM (mm) | 17.61 | <.001 | 4.03 |
| Coronal imbalance VP–DM (°) | 1.23 | .329 | 0.31 |
| Coronal imbalance VP–DM (mm) | 1.22 | .329 | 0.33 |
| Pelvic obliquity DL–DR (°) | 0.81 | .532 | 0.16 |
| Pelvic obliquity DL–DR (mm) | 0.72 | .577 | 0.11 |
| Pelvic torsion DL–DR (°) | 4.47 | .003 | 0.77 |
| Pelvic inclination (dimples) | 19.50 | <.001 | 4.02 |
| Pelvic inclination (symmetry line) | 11.59 | <.001 | 2.04 |
| Pelvis rotation | 2.39 | .067 | 0.59 |
| Cervicothoracic inflection point (ICT) | 1.73 | .167 | 0.26 |
| Kyphotic apex (KA) | 9.64 | <.001 | 1.49 |
| Thoracolumbar inflection point (ITL) | 5.60 | <.001 | 1.09 |
| Lordotic apex (LA) | 10.86 | <.001 | 1.54 |
| Lumbosacral inflection point (ILS) | 4.26 | .004 | 0.88 |
| flèche cervicale | 34.15 | <.001 | 7.20 |
| flèche lombaire | 3.58 | .010 | 0.63 |
| Kyphotic angle ICT–ITL | 42.33 | <.001 | 9.68 |
| Kyphotic angle VP–ITL | 37.21 | <.001 | 8.38 |
| Kyphotic angle VP–T12 | 26.59 | <.001 | 5.61 |
| Lordotic angle ITL–ILS | 1.78 | .162 | 0.34 |
| Lordotic angle ITL–DM | 4.31 | .004 | 0.77 |
| Lordotic angle T12–DM | 14.21 | <.001 | 2.47 |
| Vertebral rotation (RMS) | 2.14 | .094 | 0.67 |
| Vertebral rotation (max) | 1.76 | .163 | 0.49 |
| Vertebral rotation (+max) | 4.09 | .005 | 1.35 |
| Vertebral rotation (−max) | 3.73 | .009 | 0.82 |
| Vertebral rotation (amplitude) | 8.45 | <.001 | 2.10 |
| Trunk torsion | 0.92 | .470 | 0.33 |
| Apical deviation VP–DM (RMS) | 7.12 | <.001 | 1.65 |
| Apical deviation VP–DM (max) | 4.67 | .002 | 1.00 |
| Apical deviation VP–DM (+max) | 7.55 | <.001 | 1.70 |
| Apical deviation VP–DM (−max) | 3.36 | .015 | 1.00 |
| Apical deviation VP–DM (amplitude) | 8.33 | <.001 | 2.36 |
| Shoulder distance SL–SR | 6.94 | <.001 | 0.97 |
| Shoulder tilt SL–SR (°) | 1.07 | .396 | 0.23 |
| Shoulder tilt SL–SR (mm) | 1.23 | .329 | 0.27 |
| Shoulder rotation SL–SR (pelvis) | 3.05 | .023 | 0.80 |
| Shoulder rotation SL–SR (baseline) | 3.65 | .010 | 0.98 |
| *Note.* N = 1,859 for all analyses. Age was modelled using a spline with 4 degrees of freedom and adjusted for sex. P values were adjusted for multiple comparisons using the false discovery rate procedure. ΔR² represents the additional variance explained by age and is presented as a percentage. | | | |

| Table S5 Redundant parameter groups identified by Unique Variable Analysis | | |
| --- | --- | --- |
| Parameter type | Parameters Groups | Retained parameter |
| Redundant | Trunk length VP–DM (mean wTO=0.302); Trunk length T4–DM (mean wTO=0.300); Trunk length VP–SP (mean wTO=0.256) | Trunk length VP–DM |
| Redundant | Shoulder rotation SL–SR (pelvis) (mean wTO=0.573); Pelvis rotation (mean wTO=0.435); Shoulder rotation SL–SR (baseline) (mean wTO=0.365) | Shoulder rotation SL–SR (pelvis) |
| Redundant | Kyphotic angle VP–ITL (mean wTO=0.399); Kyphotic angle ICT–ITL (mean wTO=0.334); Kyphotic angle VP–T12 (mean wTO=0.323) | Kyphotic angle VP–ITL |
| Redundant | Lordotic angle ITL–DM (mean wTO=0.359); Lordotic angle ITL–ILS (mean wTO=0.321); Lordotic angle T12–DM (mean wTO=0.238) | Lordotic angle ITL–DM |
| Redundant | Vertebral rotation (max) (mean wTO=0.349); Vertebral rotation (+max) (mean wTO=0.225); Vertebral rotation (−max) (mean wTO=0.197) | Vertebral rotation (max) |
| Redundant | Apical deviation VP–DM (max) (mean wTO=0.371); Apical deviation VP–DM (−max) (mean wTO=0.242); Apical deviation VP–DM (+max) (mean wTO=0.240) | Apical deviation VP–DM (max) |
| Redundant | Shoulder tilt SL–SR (°) (max ext wTO=0.015); Shoulder tilt SL–SR (mm) (max ext wTO=0.018) | Shoulder tilt SL–SR (°) |
| Redundant | Coronal imbalance VP–DM (°) (max ext wTO=0.068); Coronal imbalance VP–DM (mm) (max ext wTO=0.069) | Coronal imbalance VP–DM (°) |
| Redundant | Pelvic obliquity DL–DR (°) (max ext wTO=0.149); Pelvic obliquity DL–DR (mm) (max ext wTO=0.131) | Pelvic obliquity DL–DR (mm) |
| Redundant | Dimple distance DL–DR (max ext wTO=0.034); DL–DR/ VP–DM (max ext wTO=0.054) | Dimple distance DL–DR |
| Redundant | Sagittal imbalance VP–DM (°) (max ext wTO=0.152); Sagittal imbalance VP–DM (mm) (max ext wTO=0.149) | Sagittal imbalance VP–DM (mm) |
| Redundant | Pelvic inclination (dimples) (max ext wTO=0.217); Pelvic inclination (symmetry line) (max ext wTO=0.230) | Pelvic inclination (dimples) |
| Redundant | Vertebral rotation (RMS) (max ext wTO=0.158); Vertebral rotation (amplitude) (max ext wTO=0.268) | Vertebral rotation (RMS) |
| Redundant | Apical deviation VP–DM (RMS) (max ext wTO=0.272); Apical deviation VP–DM (amplitude) (max ext wTO=0.232) | Apical deviation VP–DM (RMS) |
| Redundant | Thoracolumbar inflection point (ITL) (max ext wTO=0.149); Lordotic apex (LA) (max ext wTO=0.161) | Thoracolumbar inflection point (ITL) |
| Independent | Trunk length VP–SP / VP–DM ratio | Trunk length VP–SP / VP–DM ratio |
| Independent | Pelvic torsion DL–DR | Pelvic torsion DL–DR |
| Independent | Cervicothoracic inflection point (ICT) | Cervicothoracic inflection point (ICT) |
| Independent | Kyphotic apex (KA) | Kyphotic apex (KA) |
| Independent | Lumbosacral inflection point (ILS) | Lumbosacral inflection point (ILS) |
| Independent | flèche cervicale | flèche cervicale |
| Independent | flèche lombaire | flèche lombaire |
| Independent | Trunk torsion | Trunk torsion |
| Independent | Shoulder distance SL–SR | Shoulder distance SL–SR |
| Note: Parameters included in the analysis comprised those forming redundant groups with other parameters (identified via Unique Variable Analysis) and those identified as independent (showing no redundancy with any other parameter). Redundant parameters were grouped by weighted topological overlap (wTO); when a group contained three or more parameters, the parameter with the highest mean wTO within the group was retained as the most representative; when a group contained exactly two parameters, the parameter with the lower max ext wTO (i.e., greater independence from the rest of the network) was retained. Independent parameters were retained without further selection. In total, 24 parameters were retained for subsequent analyses (15 from redundant groups and 9 independent parameters).Abbreviations of anatomical landmarks: SL/SR = Shoulder Left/Right; DL/DR = Dimple Left/Right; DM = Dimple Midpoint (midpoint of the line connecting DL and DR); VP = Vertebra Prominens (C7); SP = Sacral Point; ITL = Inflection point thoracolumbar; ICT = Inflection point cervicothoracic; ILS = Inflection point lumbosacral; T4/T12 = 4th/12th thoracic vertebra; LA = Lordotic Apex. | | |
